# Supplementary material for: Stabilizing the closed SARS-CoV-2 spike trimer
Source: Nat Commun. 2021 Jan 11;12:244. doi: 10.1038/s41467-020-20321-x (PMC7801441; doi:10.1038/s41467-020-20321-x)
Supplement: Supplementary file 1 — Supplementary Information [file 41467_2020_20321_MOESM1_ESM.pdf]

Supplementary Figures

Stabilizing the Closed SARS-CoV-2 Spike Trimer

Jarek Juraszek[1#], Lucy Rutten[1#], Sven Blokland[1], Pascale Bouchier[1],
Richard Voorzaat[1], Tina Ritschel[1], Mark J.G. Bakkers[1], Ludovic L.R. Renault[2],
Johannes P.M. Langedijk[1]*

[1]Janssen Vaccines & Prevention BV, Archimedesweg 4-6, Leiden, the Netherlands

[2] NeCEN, Leiden University, Einsteinweg 55, Leiden, the Netherlands

# These authors contributed equally

* Corresponding author. e-mail: hlangedi@its.jnj.com

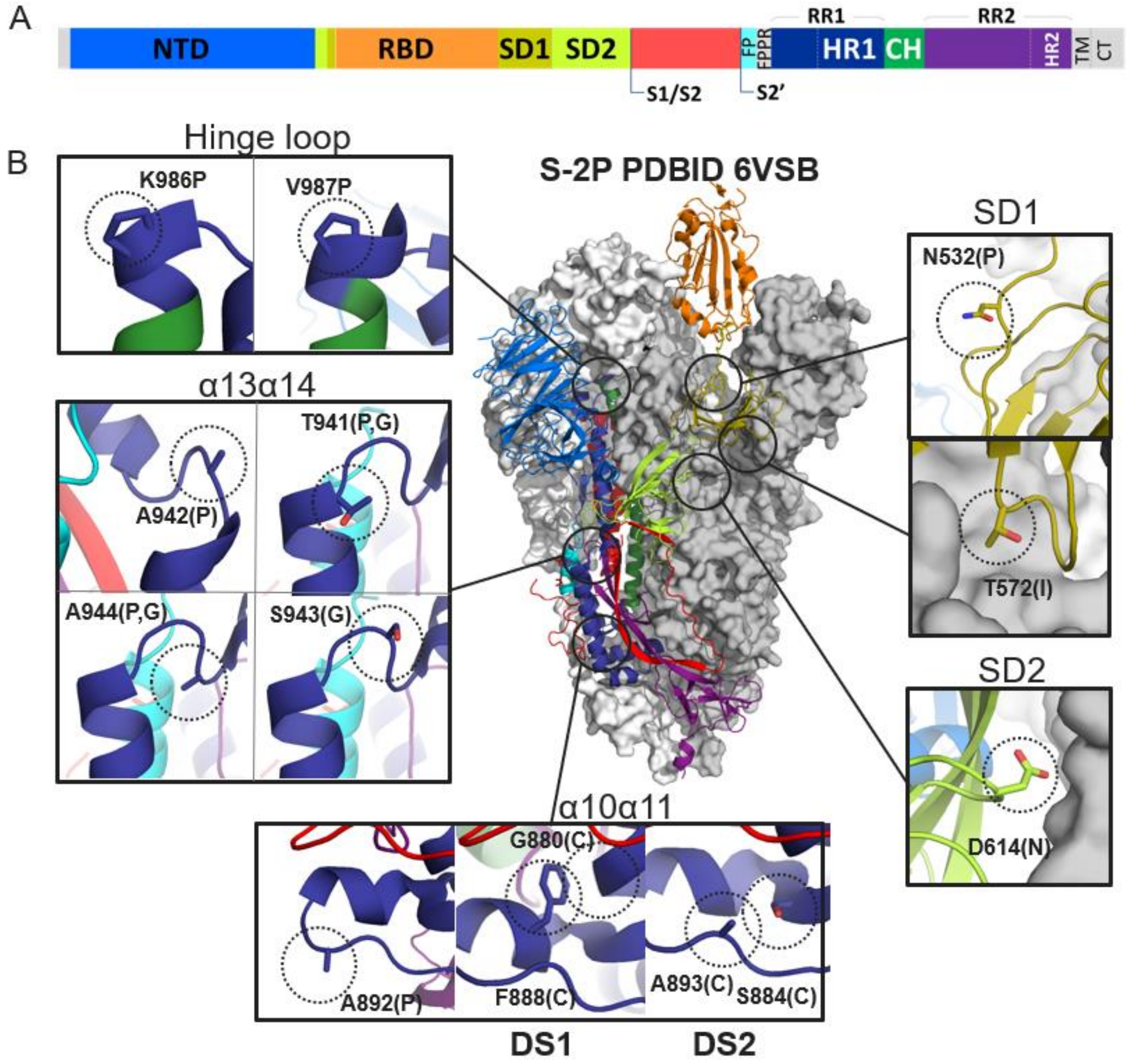

Supplementary Figure 1. Structure-based design of SARS-CoV-2 S stabilizing mutations. (A) Domains of the SARS-CoV-2 S protein with indicated cleavage sites S1/S2 and S2'. (B). Stabilizing mutations identified in the S protein grouped by structural domains. Structure of S (PDBID 6VSB) colored according to panel A for one monomer. Molecular surface of the other two monomers is plotted in grey and white. K986P and V987P, the hinge prolines stabilizing the S-2P variant are also shown. Mutations applied to the selected amino acids are indicated between brackets.

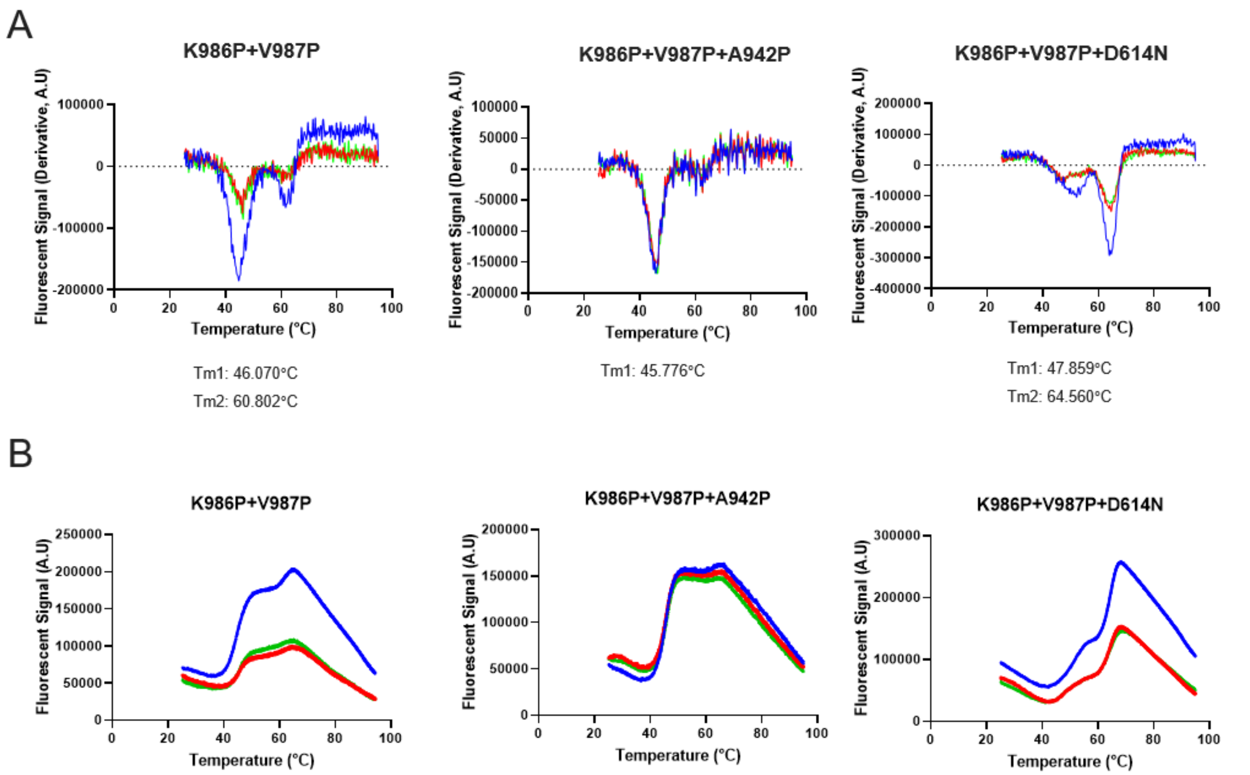

Supplementary Figure 2. Differential scanning fluorimetry. Analysis of melting temperature (Tm) using differential scanning fluorimetry of purified S protein variants. A) The first order derivatives are plotted. The experiment was done in triplicate. The Tm is determined as the lowest derivative value representing the Tm_{50} value. B) Raw fluorescence signals. Measurement 1 is shown in blue, measurement 2 in red and measurement 3 in green.

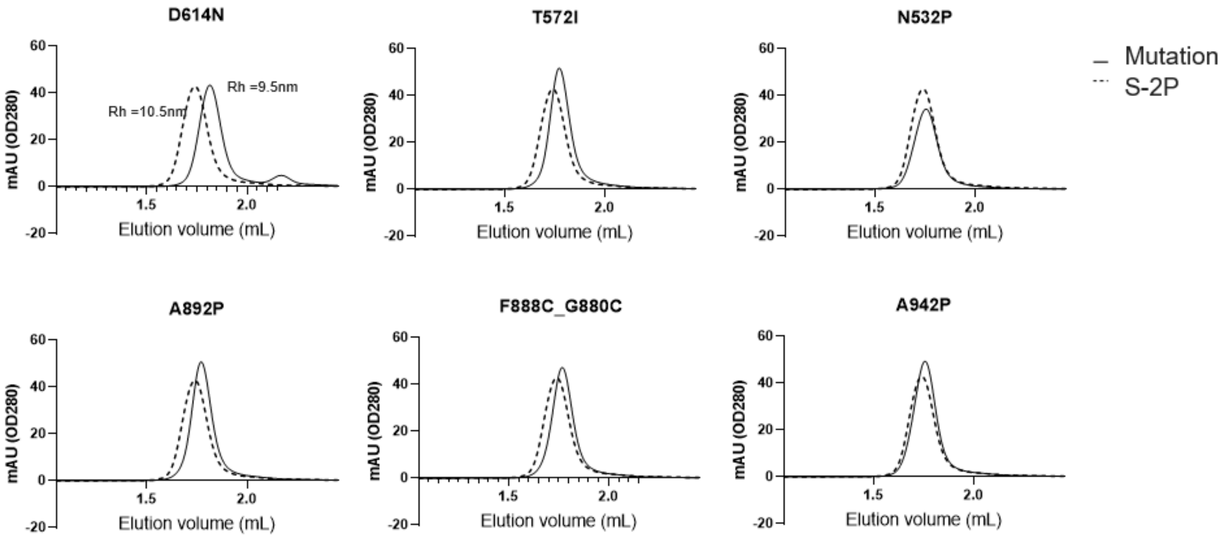

Supplementary Figure 3. Size exclusion chromatography of purified proteins. Six SEC profiles of single mutation or disulfide variants of S-2P are compared with S-2P (dotted line) on an SRT-10C SEC-500 15 cm column (Sepax Cat# 235500-4615).

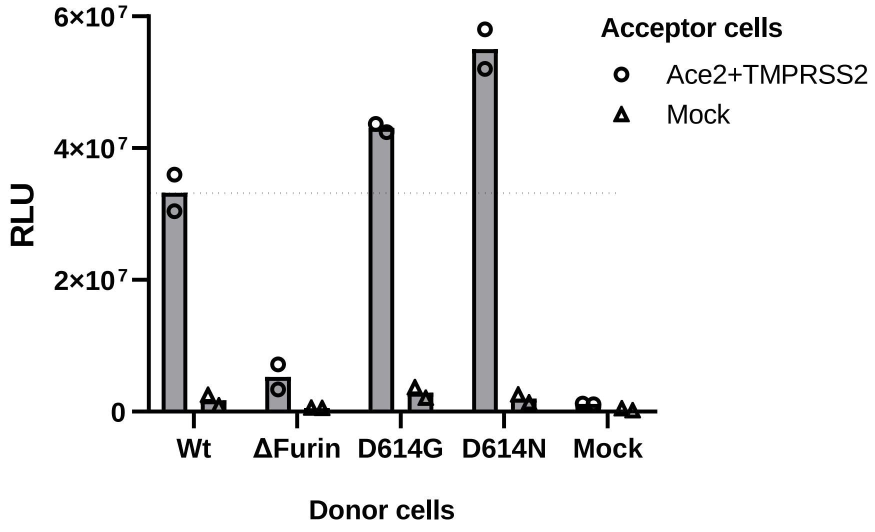

Supplementary Figure 4. Increased fusogenicity of D614N and D614G S variants. Quantitative cell-cell fusion assay in HEK293 cells using the NanoBiT split-luciferase complementation system. Donor cells were transfected with full-length wildtype S, or variants thereof, and the 11S luciferase subunit, acceptor cells were transfected with ACE2, TMPRSS2 and the PEP86 luciferase subunit, or just the PEP86 luciferase subunit ('Mock') as negative control. After 18 hr donor and acceptor cells were mixed at a 1:1 ratio and incubated for 4 hr at 37C, at which time the luciferase signal was measured. Plotted is the mean +SD of n=2 biologically independent samples examined in one experiment. The level of fusion measured for wildtype S is indicated with a dotted line.

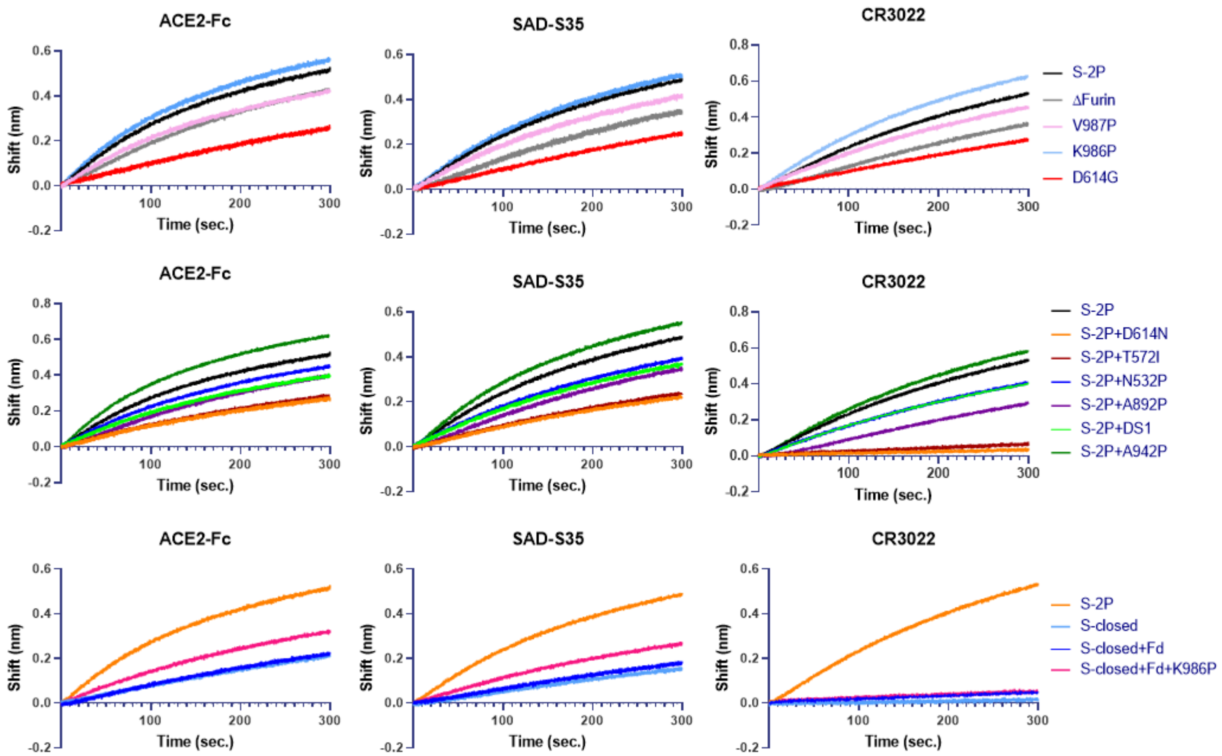

Supplementary Figure 5. BioLayer Interferometry using Octet. Bio-Layer Interferometry curves of SARS-CoV-2 S protein mutants and combinations binding to ACE2-Fc fusion, SAD-S35 and CR3022 antibodies. The curves were used to calculate the initial slope V_0 plotted in Figures 1D and 2D. In the upper three panels, S-2P and ΔFurin backgrounds are plotted in black and grey respectively. In the remaining panels, only S-2P curves are plotted for comparison.

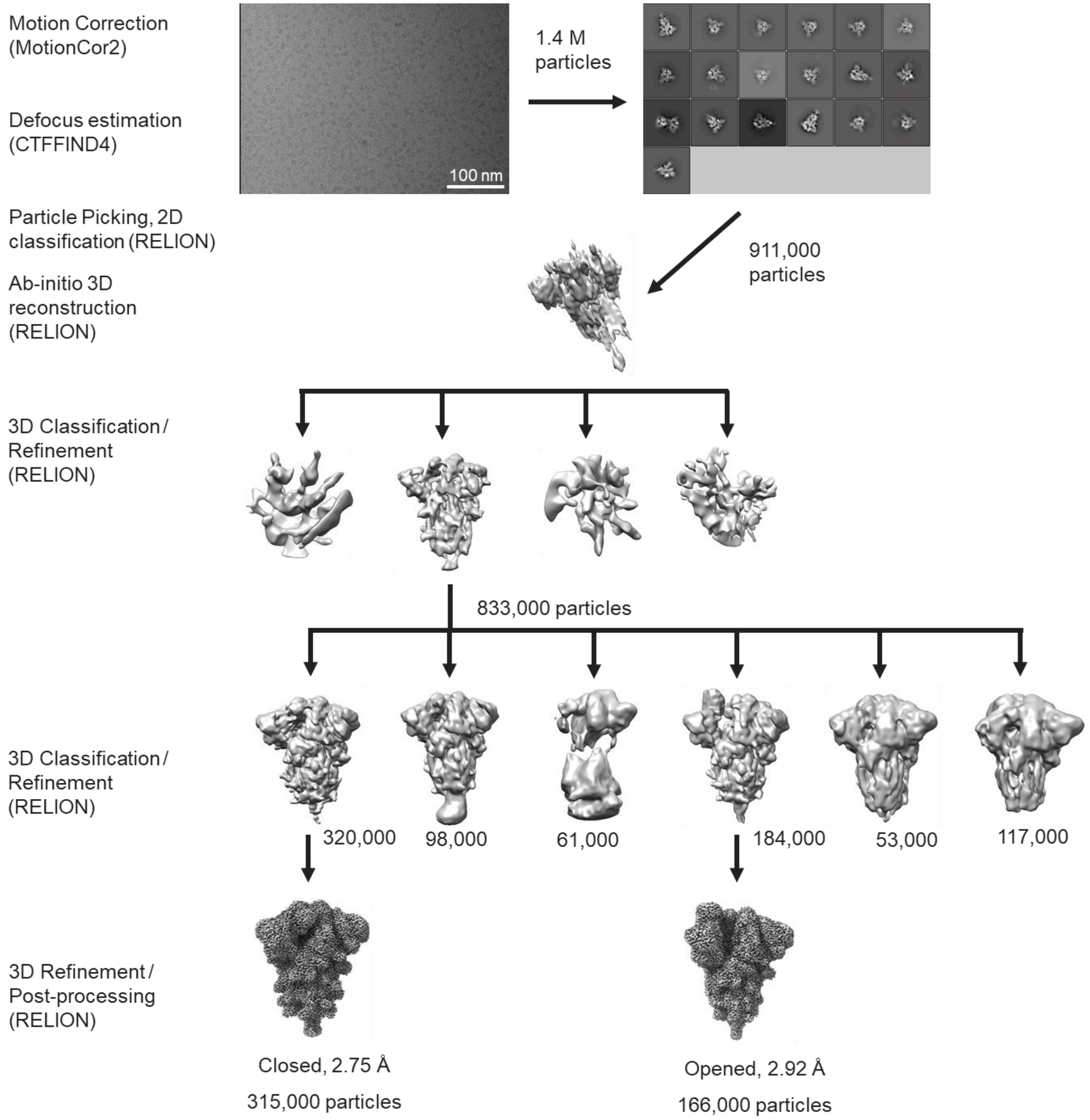

Supplementary Figure 6. Cryo-EM data processing workflow. A typical micrograph, representing approximately 75% of 9760 micrographs is shown as well as representatives 2D classes. 3D classification was performed to distinguish heterogeneity in the sample and the classes showing the highest resolution were refined.

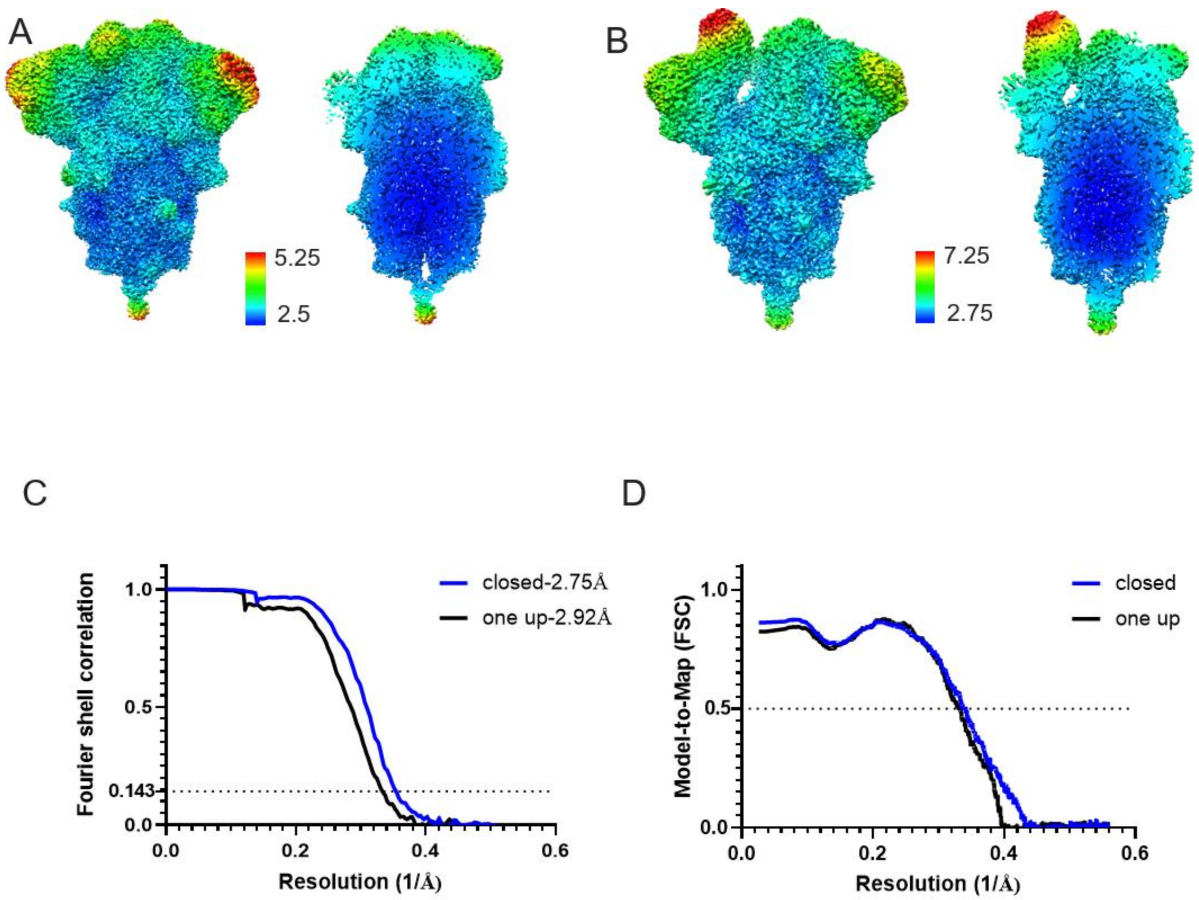

Supplementary Figure 7. Resolution assessment of cryo-EM structure: **A**) Local resolution map for closed structure (Full map, slide through, top view) **B**) Local resolution map for "one up" structure (Full map, slide through, top view) **C**) Global resolution assessment by Fourier shell correlation at the 0.143 criterion **D**) Correlations of model vs map by Fourier shell correlation at the 0.5 criterion.

Supplementary Tables

Supplementary Table 1. Thermal stabilities as measured with differential scanning calorimetry.

Constructs	Tm1 [°C]	Tm2 [°C]
ΔFurin	48.91	63.97
V987P	48.27	64.08
D614G	48.57	65.95
A944P	48.52	64.02
K986P V9787P (S-2P)	48.33	64.38
S-2P-D614N	49.49	66.23
S-2P-N532P	48.7	64.53
S-2P-T572I	49.01	65.43
S-2P-A892P	48.96	65.01
S-2P-DS1	51.76	64.61
S-2P-A942P	49.24	64.38
A892P_A942P	-	64.21
S-closed+Fd	55.02	66.32
S-closed+Fd+K986P	51.21	66.62
S-closed	51.65	66.31

Supplementary Table 2. SEC-MALS analysis

	Trimer peak				LMW species				Total
**Mutation**	**Mw (kDa)**	**Rh (nm)**	**Mass fraction (%)**	**Retention time (min)**	**Mw (kDa)**	**Rh (nm)**	**Mass fraction (%)**	**Retention time (min)**	**Recovery (%)**
**S-closed**	**465.6**	**9.165**	**89.8**	**5.319**	**ND**	**ND**	**8.7**	**5.647**	**96.7**
**S-closed+Fd**	**491**	**9.909**	**100**	**5.08**	**-**	**-**	**-**	**-**	**86.6**

Supplementary Table 3. Stability after freeze-thawing and SEC analysis

Mutation(s)	0x FT	1x FT	3x FT	5x FT
S-2P	100*	67	8	1
S-2P+D614N	100	83	43	12
S-2P+DS1	100	75	24	4
S-2P+N532P	100	72	24	3
S-2P+A892P	100	80	21	3
S-2P+A942P	100	60	13	3
S-2P+T572I	100	81	22	3
ΔFurin	100	73	10	1
D614G	100	72	21	3
A892P+A942P	100	82	29	4
S-closed+Fd+K986P	100	77	31	11
S-closed+Fd	100	74	34	8
S-closed	100	78	55	35

* Areas under the curves of the trimer peaks in SEC for non-frozen samples (0x FT) and after multiple cycles of freeze-thaw (1x, 3x, and 5x FT), normalized to their respective the non-frozen samples (100%).

Supplementary Table 4. Cryo-EM data collection

	S-closed+Fd quadruple mutant
**Magnification**	105,000
**Data collection / Final Pixel size (Å)**	0.418 / 0.836
**Voltage (kV)**	300
**Electron exposure (e^-/Å^2)**	65
**Frames**	50
**Defocus Range (μm)**	-0.6 to -2.5
**Number of movies collected**	9,760
**Particles extracted**	1,400,000
**Final (closed/opened)**	315,000 / 166,000
**Symmetry**	N/A
**Masked resolution at FSC=0.143 (Å) (closed/opened)**	2.75/2.92
**Map sharpening B-factor (Å^2) (closed/opened)**	-52/-51
**EMDB**	EMD-11639 / EMD-11719

Supplementary Table 5. Model refinement and validation statistics

		Closed trimer	One up trimer
**PDB**		7A4N	7AD1
**Model compositions**			
	Amino Acids	2889	2841
	Glycans	21	35
**Mean B factors (Å^2)**			
	Amino Acids	94.53	107.22
	Glycans	75.34	112.75
**R.m.s. deviations**			
	Bond lengths (Å)	0.008	0.016
	Bond angles (˚)	1.057	1.260
**Validation**			
	MolProbity score	1.53	1.95
	Clashscore	5.08	8.72
	Rotamers outliers (%)	0.20	0.00
	EMRinger score	3.64	3.51
**Ramachandran plot**			
	Favored (%)	96.14	92.15
	Allowed (%)	3.86	7.85
	Outliers (%)	0.00	0.00
